# Desmoplastic Small Round Cell Tumor of the Head and Neck: A Potential Diagnostic Pitfall

**DOI:** 10.7759/cureus.30475

**Published:** 2022-10-19

**Authors:** Ebtihal Alharbi

**Affiliations:** 1 Pathology, Faculty of Medicine In Rabigh, King Abdulaziz University, Rabigh, SAU

**Keywords:** soft tissue sarcoma, ewsr1-wt1 gene fusion, sinonasal tract, eye, salivary glands, head and neck, sarcoma, dsrct, desmoplastic small round cell tumor

## Abstract

Primary desmoplastic small round cell tumor (DSRCT) in the head and neck region is extremely rare. There is limited information about its clinicopathological characteristics, prognosis, and treatment modalities. The purpose of this study is to provide a comprehensive review of DSRCT occurring primarily in the head and neck, to demonstrate its peculiar morphology and immunohistochemical expression, and to address the differential diagnoses. A total of 25 cases were collected after a thorough review of the relevant literature. DSRCT was most frequently reported in the major salivary glands, followed by the eyes. Furthermore, some cases were misinterpreted as poorly differentiated carcinoma, Ewing sarcoma, and olfactory neuroblastoma. Diagnosing DSRCTs in the head and neck can be very challenging due to their rarity in this location, overlapping morphology, and immunohistochemistry. In these cases, following a systemic approach helps to solve diagnostic problems.

## Introduction and background

Mesenchymal tumors of the head and neck are very rare, accounting for 35% of cases in children and 5-15% of cases in adults [1-3]. Most of them are benign, whereas malignant tumors constitute only 1% [4]. The majority of these tumors originate in the parotid glands, scalp, forehead, and neck; however, they can arise in any anatomical location. Patients usually complain of mass, which may be accompanied by pressure feelings and pain. Some patients report sinonasal symptoms, such as nasal blockage, discharge, and bleeding, whereas others complain of visual impairment. Even though mesenchymal tumors in the head and neck are often smaller than those in other anatomic regions, complete excision with free margins might be challenging and the recurrence rate may be significant [5].

Desmoplastic small round cell tumor (DSRCT) is a rare malignant mesenchymal tumor composed of small round blue cells and desmoplastic stoma in the background. Gerald and Rosai were the first to describe this tumor as a distinct entity in 1989 [6]. The cellular origin of this tumor is uncertain. It predominantly affects young men, but anyone can be affected. The mean age is 22 years [7]. The presenting symptoms vary depending on the location of the tumor. Most commonly, the tumor originates in the abdomen and/or pelvis and the symptoms include abdominal mass, pain, constipation, and urinary retention. Other sites, such as the posterior cranial fossa, lung, kidney, hand, and pleura have been described [7]. Diagnosing DSRCT in the head and neck is extremely unusual, and only a few cases have been reported in the literature; some of them were misdiagnosed as poorly differentiated carcinoma or Ewing sarcoma [8,9]. This article aims to review the published cases of DSRCT in the head and neck, highlight its unusual morphology, and discuss the differential diagnoses.

## Review

Materials and methods

The English literature was extensively searched in PubMed and Google Scholar. The titles and abstracts were searched using the following terms: (desmoplastic small round cell tumor) AND (head and neck OR eye OR orbit OR ear OR sinonasal tract OR salivary gland OR mandible OR maxilla OR oral cavity OR larynx OR pharynx OR extra-abdominal). Twenty-five cases were collected; all of them were case reports and case series. Inclusion criteria were limited to primary DSRCTs originating in the head and neck. Exclusion criteria were primary intracranial tumors and metastatic tumors that involved the head and neck or cervical lymph nodes.

Result

The vast majority of the cases occurred in males. The median age was 25 years. The major salivary gland was the most frequently reported location, with seven cases found in the submandibular gland and five in the parotid gland. The eye came next, with five cases reported. On histologic examination, the majority of cases exhibited the typical morphology of well-defined nests of monomorphic small round blue cells separated by vascularized desmoplastic stroma. DSRCT has a unique triphenotypic immunohistochemical profile, as it expresses epithelial markers including keratin and epithelial membrane antigen (EMA), myogenic markers including desmin, and nerve sheath-associated antigens including neuron-specific enolase (NSE), CD57, and neuroendocrine markers. It is critical to detect the distinctive* EWSR1-WT1* gene rearrangement in order to establish the diagnosis. Fourteen cases had the typical immunohistochemical profile, and molecular studies confirmed the diagnosis of all of them except for one case [10]. Certain cases exhibited atypical immunohistochemical expressions, such as P63 and P40 positivity or desmin negativity [8,11-13]. The treatment for DSRCT is surgical resection followed by chemotherapy with or without radiotherapy. Complete surgical resection can be exceedingly difficult, especially in the head and neck, due to its infiltrative nature and proximity to vital structures. The patients were followed up for a period ranging from one to 48 months; four patients died of the disease or its complications [10,13-15], six patients had lymph nodes metastases [9-11,14-16], three patients lived with recurrence [8,16,17], and one lived with distant metastases [9]. Due to the rarity of this tumor in the head and neck and its unique immunohistochemical profile, four cases were misdiagnosed as Ewing sarcoma/peripheral primitive neuroectodermal tumor, olfactory neuroblastoma, and poorly differentiated carcinoma [8,9,18]. Table 1 shows a summary of all reported cases.

**Table 1:** Summary of reported cases of primary desmoplastic small round cell tumor of the head and neck (n=25) NS: not specified; FOD: free of disease; AWD: alive with disease; DOD: died of disease; DOC: died of disease complications; Syn: synaptophysin; NSE: neuron specific enolase; CK: cytokeratin; EMA: epithelial membrane antigen; Vim: vimentin; SMA: smooth muscle actin; M: male; RT-PCR: reverse transcription-polymerase chain reaction; FISH: fluorescence in situ hybridization;

Reference	Age (year)	Sex	Presenting symptom	Location	Size (cm)	Positive immunostains	Molecular result	Lymph node metastasis	Treatment	Margin status	Outcome (month)
Wolf et al. [19]	4	M	Slowly growing mass	Parotid gland	5	Desmin, EMA, CK, NSE	RT-PCR: *EWS-WT1* gene fusion	No	Chemo, resection, and radio	Positive	10, FOD
Hill et al. [20]	5	M	NS	Parotid gland	NS	Desmin, EMA, CK, NSE, WT1, Vim	RT-PCR: *EWS-WT1 *gene fusion	NS	NS	NS	NS
Lae et al. [12]	6	M	Weight loss and headache	Scalp soft tissue	NS	AE1/AE3, CAM5.2, NSE, WT1	NS	NS	Resection and chemo	NS	13, AWD
Finke et al. [21]	16	F	Chronic sinusitis	Frontal, ethmoidal and sphenoid sinus with brain and skull base extension	Fragments measured in aggregate 8.5	Desmin, CAM5.2, CK, NSE, Vim	RT-PCR: *EWS-WT1 *gene fusion	NS	Resection, chemo, radio	NS	26 , FOD
Yoon et al. [22]	16	M	Visual symptoms	Eye	2.5	Desmin, CD99, CK, NSE	FISH: *EWSR1 *breakapart	NS	Resection, radio	NS	> 11 FOD
Cho et al. [14]	16	M	Mass	Submandibular gland	4	Desmin, CK, NSE	RT-PCR: *EWS-WT1 *gene fusion	Yes	Resection, radio, chemo	NS	25, DOD
Gorjon et al. [23]	17	M	Mass, pain	Submandibular gland	5	CAM 5.2, EMA, vim, desmin, progesterone receptors, C-Kit, B-catenin (cytoplasmic)	RT-PCR: *EWS-WT1* gene fusion	No	Resection	Negative	10, FOD
Kupeli et al. [10]	18	M	Mass	Mandible	NS	CK, NSE, desmin, vim	Not done	Yes	Chemo, radio	NS	22, DOC
Rekhi et al. [8]	21	M	Nasal blockage, discharge	Maxilla	4.5	Vim, CD99, FLI1, CD56	RT-PCR: *EWS-WT1 *gene fusion	NS	Resection, chemo, radio, treated as Ewing sarcoma	NS	3, AWD
Yin et al. [24]	23	M	Mass	Submandibular gland	4	Desmin, CK, NSE , P53	FISH: *EWSR1* breakapart, RT-PCR: *EWS-WT1 *gene fusion	NS	Resection, chemo, radio	NS	7, FOD
Pang et al. [15]	24	M	Mass	Submandibular gland	5	Desmin, EMA, CK, WT1, CD56	FISH: *EWSR1 *rearrangement, RT-PCR: *EWS-WT1* gene fusion	Yes	Resection	Negative	1 DOC
Lopez et al. [18]	25	M	Nasal blockage, bleeding	Ethmoid sinus	8	AE1/AE3, CK8/CK18, EMA, desmin, Vim, NSE, WT1	FISH: *EWSR1* breakapart, RT-PCR: *EWS-WT1* gene fusion	NS	Resection, radio	Negative	29, FOD
Khachaturov et a.l [25]	25	M	Pain and swelling	Calvarium	NS	Desmin, CD99, CK	RT-PCR: *EWS-WT1 *gene fusion, fusion of *EWSR1* exon 10 to *WT1 *exon 8 by sequencing	NS	Chemo, radio	NS	5, FOD
Cobanoglu et al. [26]	26	M	Swelling	Eye	3.2	Desmin, WT1, NSE, CD99, CAM 5.2	*EWSR1-WT1 *fusion	NS	Chemo, concurrent proton therapy, resection	Negative	12, FOD
Wang et al. [27]	27	M	Mass	Eye	NS	Desmin, CD99, NSE, Syn, SMA	FISH: *EWSR1* rearrangement	NS	Resection	Negative	12, FOD
He et al. [28]	30	M	Painful mass	Eye	1.5	Desmin, CD99, CD56, NSE, Syn, SMA, Vim	FISH: *EWSR1* rearrangement	No	Resection	NS	12, FOD
Xu et al. [29]	32	M	Ear discharge, facial palsy	Middle ear mastoid	NS	NS	NS	NS	Resection, chemo, radio	NS	48, FOD
Tao et al. [13]	36	F	Eye epiphora, nasal bleeding	Nasal cavity and ethmoid sinus	NS	CD56, Vim, WT-1	NS	NS	Resection, chemo, radio	NS	2, DOD
Hatanaka et al. [30]	36	M	Mass	Parotid gland	2.7	Desmin, EMA, CK, WT1, CD56 , Vim	FISH: *EWSR1* breakapart, RT-PCR: *EWS-WT1 *gene fusion	Negative	Resection, radio	Positive	36, FOD
Cai et al. [11]	38	M	Mass	Parotid gland	5	AE1/AE3, p40, p63, desmin, GATA3 (weak)	FISH: *EWSR1* rearrangement, NGS: *EWSR1-WT1* gene fusion	Yes	Resection, chemo, radio	NS	5, FOD
Ninchritz-Becerra et al. [16]	41	M	Mass	Parotid gland	3	AE1/AE3, desmin,	FISH: *EWSR1* rearrangement	Yes	Chemo, resection	NS	14, AWD
Li et al. [17]	49	M	Mass	Submandibular gland	3	CK, EMA, desmin, Vim, CD56, Syn, CD99, NSE, Fli-1	FISH: *EWSR1* breakapart	NS	Resection, chemo, radio	Negative	21, AWD
Sun et al. [9]	55	M	Visual symptoms	Eye	2.4	AE1/AE3 (3/3), desmin (3/3), Vim (2/2), CD99 (2/2), Syn (1/3), NSE (1/1), EMA (1/1), retained INI-1 (1/1)	FISH: *EWSR1* rearrangement in all of the cases	NS	Resection, chemo, radio	NS	14, FOD
	59	M	Mass	Submandibular gland	Two masses one 3.8 and the other one 1			NS	Resection, chemo	NS	5, AWD
	61	M	Mass	Submandibular gland	2.5			Yes	Resection, chemo	NS	8, FOD

Discussion

**Figure 1:**
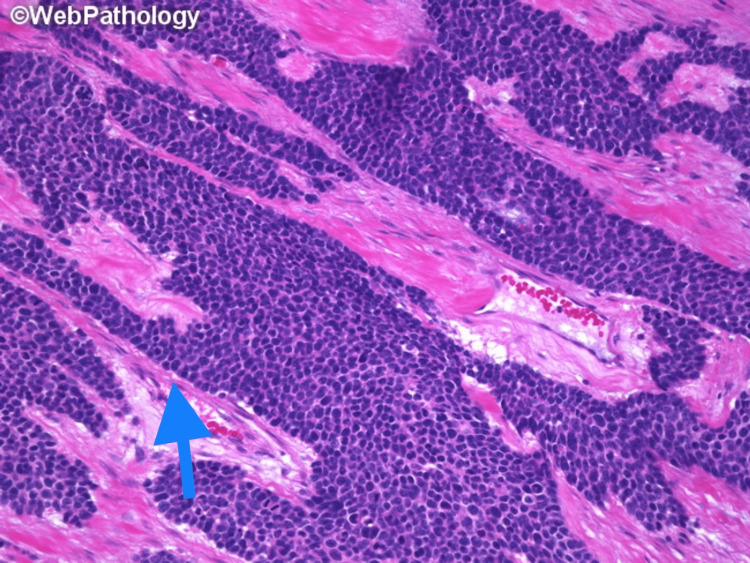
Anastomosing trabeculae of small round blue cells (arrow) and vascularized collagenous stroma in the background Used with permission from Dharam M. Ramnani, MD, Richmond, Virginia, USA, webpathology.com [31]

**Figure 2:**
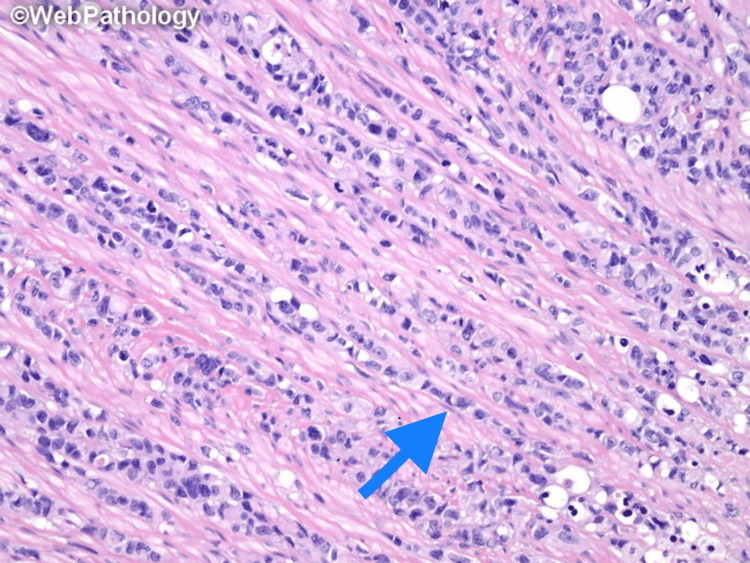
Small round blue cells with mild to moderate pleomorphism arranged in a single-file pattern (arrow) Used with permission from Dharam M. Ramnani, MD, Richmond, Virginia, USA, webpathology.com [32]

**Figure 3:**
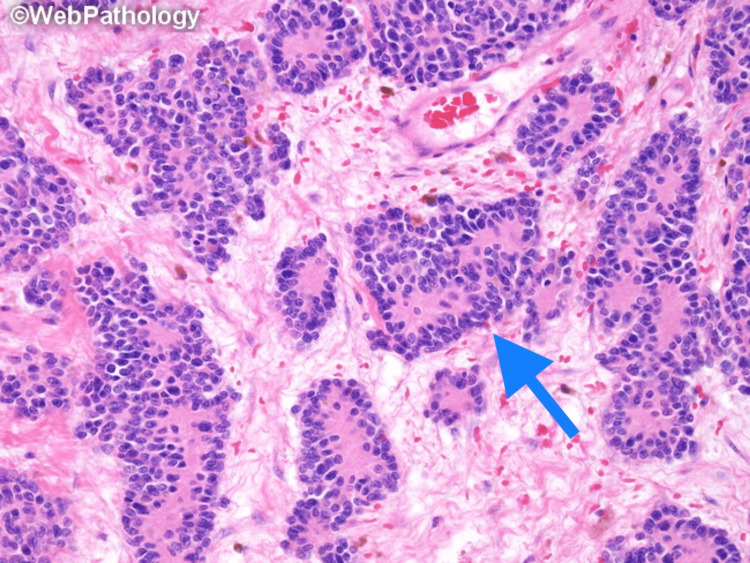
Monomorphic small round blue cells arranged in Homer Wright-like rosettes (arrow), and vascularized collagenous stroma in the background Used with permission from Dharam M. Ramnani, MD, Richmond, Virginia, USA, webpathology.com [33]

DSRCT is very aggressive, and many patients have metastatic disease at the time of diagnosis. Macroscopically, it is grey-tan, lobulated, firm, and solid. Hemorrhage, necrosis, and degenerative changes have been observed in some cases. The classic histologic morphology consists of round to oval cells with scant pale to eosinophilic cytoplasm, hyperchromatic monomorphic nuclei, and inconspicuous nucleoli. Necrosis and pleomorphism can be seen in some areas. The tumor cells are arranged in several patterns, including nested, trabecular, and single-file, as shown in Figure 1 and Figure 2 [31,32]. These structures are surrounded by a stromal band consisting of collagen, fibroblasts, and myofibroblasts. It is important to note that some of the histologic features of DSRCT may overlap with other tumors and lead to misinterpretation. Large cytoplasmic vacuoles, the appearance of a signet ring, and papillae may be focally observed and mistaken for carcinoma. Homer Wright-like rosettes that resemble olfactory neuroblastoma and Ewing sarcoma, as shown in Figure 3 [33]. Rhabdoid cells can be confused with rhabdomyosarcoma cells. Some tumors have elongated nuclei and the differential diagnosis of spindle cell lesions should be considered [34]. By immunohistochemistry, the tumor expresses epithelial, myogenic, and neural markers. Desmin is found in up to 90% of cases and exhibits a perinuclear dot-like pattern. Smooth muscle actin (SMA) and muscle-specific actin (MSA) are rarely expressed, whereas other myogenic markers such as MyoD1 and myogenin are negative [35]. Almost all DSRCTs are positive for epithelial markers, either cytokeratins, EMA, or both [7]. CK5/6 and CK20 are typically negative [35]. NSE and CD57 are expressed in 82% and 49% of cases, respectively [35]. CD99 can show cytoplasmic positivity along with NB48; however, the usefulness of these markers is limited since they are also positive in Ewing sarcoma and neuroblastoma. WT1 is positive because of the *EWSR1-WT1 *gene rearrangement. By molecular study, *EWSR1-WT1* gene rearrangement can be detected by fluorescence in situ hybridization (FISH) or reverse transcription-polymerase chain reaction (RT-PCR) [7].

Small round blue cell tumors are a group of tumors that share several morphologic characteristics, including dense cellularity, scant cytoplasm, and round, oval, or angular nuclei. It might be challenging to interpret this histologic pattern, particularly in small biopsies. The following factors must be considered when examining these tumors: (i) clinical and radiological information, (ii) histological architecture and cellular details, (iii) careful immunohistochemical interpretation and familiarity with immunophenotypic overlap, and (iv) molecular analysis. The differential diagnosis for head and neck small round blue cell tumors includes: (i) carcinomas such as squamous cell carcinoma, nuclear protein of the testis (NUT) midline carcinoma, SWI/SNF (swItch/sucrose non-fermentable)‐related matrix‐associated actin‐dependent regulator of chromatin subfamily B member 1 (SMARCB1)-deficient carcinoma, sinonasal undifferentiated carcinoma (SNUC), a solid variant of adenoid cystic carcinoma, and neuroendocrine small cell carcinoma, (ii) soft tissue tumors such as Ewing sarcoma family of tumors, DSRCT, rhabdomyosarcoma, and poorly differentiated synovial sarcoma, (iii) neuroectodermal/melanocytic tumors such as olfactory neuroblastoma and mucosal malignant melanoma, and (iv) hematolymphoid tumors [36,37].

Carcinomas

Squamous cell carcinoma is the most common malignancy in the upper aerodigestive tract, especially in the oral cavity and larynx [38]. Poorly differentiated nonkeratinizing squamous cell carcinoma can overlap with other small round blue cell tumors. Recognizing better-differentiated areas or dysplastic/in situ components in the surface epithelium helps to reach the diagnosis. By immunohistochemistry, the tumor is positive for CK5/6, P63, and P40 while negative for neural markers [39].

Basaloid squamous cell carcinoma (BSCC) is an uncommon, aggressive variant that tends to arise in the head and neck region, particularly the base of the tongue, supraglottic larynx, and pyriform sinus [40]. Squamous cell carcinoma with basaloid morphology and associated with high-grade human papillomavirus (HPV) has a better prognosis [41]. Histologically, the tumor is connected to the surface epithelium, which has high-grade dysplasia or squamous cell carcinoma. It is composed of basaloid cells with scant cytoplasm, hyperchromatic nuclei, invisible nucleoli, pleomorphism, and frequent mitotic activity. The tumor cells are arranged in nests and lobules with peripheral palisading and a thick basement membrane. Comedonecrosis is commonly seen. The tumor is immunohistochemically positive for pancytokeratin, EMA, P63, P40, and low molecular weight cytokeratin. NSE is occasionally positive, whereas chromogranin and synaptophysin are typically negative [42].

NUT midline carcinoma is an aggressive high-grade carcinoma that arises in the midline sites and usually affects young individuals. Histologic examination shows undifferentiated primitive cells with abrupt squamous differentiation. This tumor exhibits diffuse nuclear positivity (>50%) for NUT monoclonal antibody and stains positively for pancytokeratin, EMA, p63, and p40 [43]. INI1 nuclear expression is retained. Molecular tests for NUT rearrangement are fundamental to confirm the diagnosis. When examining a tumor with such morphology, it is important to consider adamantinoma-like Ewing sarcoma because it shows squamous differentiation and is diffusely positive for keratins, including high molecular weight keratins [44]. Therefore, immunostains for NKX2.21 and Fli1 and molecular tests for *EWSR1-FLI1 *gene rearrangement are needed.

SNUC is a highly aggressive carcinoma of the sinonasal tract that lacks glandular, squamous, and neuroendocrine differentiation. A thorough histologic examination and careful immunohistochemical interpretation are required since this tumor is diagnosed by exclusion. Microscopically, the tumor cells are monotonous and polygonal, with abundant cytoplasm, distinct cell borders, vesicular chromatin, and prominent nucleoli. The tumor cells are organized into sheets, lobules, and trabeculae. By immunohistochemistry, SNUC is positive for pancytokeratin and low molecular weight cytokeratin but usually not high molecular weight cytokeratin [45]. Markers for squamous and neuroendocrine cells may be focally positive, but diffuse positivity should not be seen [46].

A recently described tumor of the sinonasal tract, SMARCB1-deficient carcinoma, is characterized by the inactivation of the *SMARCB1 (INI1) *tumor-suppressor gene [47]. This tumor, like other SMARCB1-deficient tumors, exhibits rhabdoid or plasmacytoid cytomorphology. Immunohistochemical studies show this tumor is typically positive for cytokeratins, negative for nuclear INI1 staining, and variable for P40 and neuroendocrine markers [46,47].

Neuroendocrine small cell carcinoma has been described in numerous head and neck locations, including salivary glands, larynx, nasal cavity, paranasal sinuses, oral cavity, and pharynx. Microscopic examination reveals sheets of small cells with scant cytoplasm, dark chromatin, nuclear molding, increased mitotic figure, apoptosis, and necrosis. The tumor is immunohistochemically positive for pancytokeratin, low molecular weight cytokeratin, and neuroendocrine markers while negative for high molecular weight cytokeratin. CK20 is positive in the Merkel cell type and negative in the pulmonary type [48]

Adenoid cystic carcinoma, particularly the solid variant, can mimic small round blue cell tumors. Identifying areas of cribriform differentiation with distinct ductal and basal cell populations is a useful histological clue. Immunostains confirm the existence of two cell populations, with ductal cells expressing CK7, c-kit, and EMA, and basal cells expressing S100, p63, p40, SMA, and calponin [49,50].

Soft Tissue Tumors

Rhabdomyosarcoma is a malignant soft tissue tumor with skeletal muscle differentiation. It is one of the most prevalent soft tissue sarcomas in children and adolescents and commonly develops in the head and neck region, where 26% of cases occur. Several subtypes are identified in the head and neck including embryonal, and alveolar. On a histological level, rhabdomyosarcoma consists of small, round blue cells that may be organized into a solid sheet or alveolar pattern. Rhabdomyoblasts are primitive cells with cross-striated cytoplasm. Although rhabdomyoblasts are a useful clue that suggests rhabdomyosarcoma, it is not totally specific [51]. By immunohistochemistry, the tumor expresses desmin, myogenin, and myoD1. It is important to note that alveolar rhabdomyosarcoma can express focally neuroendocrine markers and cytokeratins, which might cause confusion. Molecular tests for *PAX3-FOX01* or *PAX7-FOX01* gene fusions are essential to confirm the diagnosis of alveolar rhabdomyosarcoma [51].

Ewing sarcoma family of tumors is a high-grade malignant tumor characterized by *EWSR1* gene rearrangement. It most commonly affects young patients with a slight male predilection. Histologically, the tumor is composed of sheets of primitive monomorphic cells with clear cytoplasm, round nuclei, and vesicular chromatin. Strong and diffuse membranous positivity for CD99 is characteristic [52]. Additionally, both FLI-1 and NKX2.2 have nuclear positivity. In some cases, neuroendocrine markers such as synaptophysin and chromogranin, as well as epithelial markers such as low molecular-weight cytokeratin, can be positive. High molecular-weight cytokeratin and P63 are strongly positive in adamantinoma-like Ewing sarcoma [44]. Molecular studies are required to detect *EWSR1 *gene rearrangement [44,52].

Poorly differentiated synovial sarcoma (PDSS) is a malignant soft tissue tumor with uncertain differentiation that typically affects young adults. It accounts for 2-29% of soft tissue tumors of the head and neck. Histologically, it can be seen in three patterns, one of them is a small round blue cell pattern. Hemangiopericytoma-like blood vessels are frequently seen in the background. Intracytoplasmic hyaline inclusions mimicking rhabdomyoblast might be seen. Cytokeratin immunostaining is typically focal, and high molecular weight cytokeratin has been found to be more sensitive [53]. CD 99 is frequently positive whereas CD34 is usually negative. TLE-1 (transducin-like enhancer of split 1) is a highly sensitive but not specific marker [54]. Gene fusions of *SS18/SSX1* or *SS18/SSX2* can be detected by FISH or RT-PCR [53,54].

Neuroectodermal/Melanocytic Tumors

Olfactory neuroblastoma (ONB) is derived from the olfactory neuroepithelium in the cribriform plate region. Low-grade tumors are characterized by nests of tumor cells separated by vascular stroma. The tumor cells are monomorphic with fine chromatin, invisible nucleoli, abundant neuropil, and Homer Wright pseudorosettes. In addition, spindle or stellate cells, known as sustentacular cells, are present at the lobules' periphery. High-grade tumors exhibit diffuse growth patterns with marked nuclear pleomorphism, increased mitotic figure, necrosis, Wintersteiner rosettes, and minimal neuropil. By immunohistochemistry, the cells in the lobules are positive for synaptophysin, chromogranin, and CD56, while negative for CK. Sustentacular cells are positive for S100 and GFAP [55].

Mucosal malignant melanoma is an aggressive tumor that most commonly arises in the sinonasal mucosa. The histologic characteristics are variable; tumor cells may exhibit epithelioid, spindled, or small round blue cell morphology. Identifying in situ components supports the diagnosis. The tumor is immunohistochemically positive for S100, SOX10, HMB45, and Melan-A. In rare cases, tumors exhibit aberrant expression of cytokeratin, desmin, chromogranin, synaptophysin, and calponin [56,57].

Hematolymphoid Tumors

Extranodal natural killer (NK)/T-cell lymphoma is an aggressive lymphoma that arises from activated NK cells and is associated with the Epstein-Barr virus (EBV). Angiocentric and angioinvasive growth patterns with associated fibrinoid necrosis are very important histologic clues. By immunohistochemistry, the tumor is positive for CD56, CD57, cytoplasmic CD3, TIA1, granzyme, and perforin while negative for surface CD3, CD4, CD5, and CD8 [58].

## Conclusions

Small round blue cell tumors of the head and neck have significant histological and immunohistochemical overlap; therefore, it is essential to consider a broad differential diagnosis that includes epithelial, mesenchymal, neuroendocrine, and lymphoid tumors. Following a comprehensive approach that incorporates clinical and radiological information, histologic features, immunohistochemical profile, and molecular tests is necessary to reach the diagnosis. Awareness of the uncommon presentation, anatomical location, histologic features, and immunohistochemical profile of DSRCT aids the practicing pathologist in avoiding misdiagnosis.
